# Effect of blood flow restriction training on pressure pain threshold and hand function among adults with persistent neck pain: A study protocol for a randomized controlled trial

**DOI:** 10.12688/f1000research.140084.2

**Published:** 2024-05-31

**Authors:** Mohammad Sidiq, Aksh Chahal, Nitesh Bansal, Sajjad Alam, Rituraj Verma, Krishna Reddy Vajrala, Jyoti Sharma, Sumera Khan, Yamini Sharma, Balamurugan Janakiraman, Richa Hirendra Rai, Nitesh Malhotra

**Affiliations:** 1 Department of Physiotherapy, Galgotias University, Greater Noida, Uttar Pradesh, 203201, India; 2 Jindal School of Public Health & Human Develeopment, O P Jindal Global University, Sonipat, Haryana, 131001, India; 3 Faculty of Physiotherapy, Madhav University, Abu Road, Rajasthan, India; 4 SRM College of Physiotherapy, SRM Institute of Science and Technology (SRMIST), Kattankulathur, Chennai, Tamil Nadu, 6032032, India; 5 Physiotherapy, Delhi Pharmaceutical Sciences and Research University, New Delhi, Delhi, 110017, India; 6 Department of Physiotherapy, School of Allied Health Science, Manav Rachna International Institute of Research and Studies, Faridabad, Haryana, 121003, India

**Keywords:** Blood Flow Restriction Training, Persistent Neck Pain, Pressure Pain threshold, Hand grip strength, hand function

## Abstract

**Background:**

Persistent neck pain is a prevalent musculoskeletal condition that affects the quality of life and functional abilities of individuals. Blood Flow Restriction Training (BFRT) is a novel therapeutic approach that involves restricting blood flow to exercising muscles to enhance strength and function. However, limited research has been conducted on the effects of BFRT on pressure pain threshold and hand function in adults with persistent neck pain. This randomized controlled trial aims to investigate the potential benefits of BFRT as a treatment intervention for this population.

**Methods:**

This study will be a prospective 1:1 allocation, parallel group active controlled trail conducted at Physiotherapy Department, Galgotias University. The trial was prospectively registered with the Clinical Trial Registry India CTRI/2023/06/053439. Informed consent will be obtained from all the participants who are eligible to be included in the study. A total of 110 patients with persistent neck pain will be randomly allocated into two groups. The BFRT group will receive supervised training sessions three times a week for eight weeks, performing low-load resistance exercises with blood flow restriction applied using personalized cuff pressure. The control group will receive standard care for neck pain, which may include general advice, manual therapy, and/or home exercises without BFRT. The primary outcome measures will be the pressure pain threshold, assessed using a pressure Algometer, and hand function, evaluated using standardized tests such as Hand Grip Strength and Purdue Peg board Test.

**Results:**

The data obtained will be analyzed using appropriate statistical methods, and the significance level will be set at p<0.05.

**Conclusion:**

This trial will contribute valuable contribution highlighting the potential benefits of BFR training in improving pressure pain threshold and hand function in adults with persistent neck pain.

## Introduction

Persistent neck pain is a prevalent and debilitating condition that affects a significant number of adults worldwide. ^1^ The burden of this condition on individuals’ quality of life and productivity underscores the need for effective and innovative interventions to alleviate pain and improve functional outcomes. ^2^ Exercise serves as the principal non-pharmacological approach to ameliorate function in musculoskeletal disorders that cause pain. Resistance training regimen for the muscles has been shown to increase pain threshold and improve function, thereby alleviating discomfort. In order to induce muscle strength gain, the American College of Sports Medicine (ACSM) advises a resistance load of 60% to 70% of one repetition maximum (RM). At the same time, studies have reported that resistance training with increased loads leads to heightened stress levels, consequently resulting in increased pain, reduced patient adherence, and potential condition deterioration. Therefore, researchers have turned their attention towards developing a suitable resistance training program that is less taxing and more tolerable for individuals suffering with painful neck conditions. BFRT is one method that permits a pairing with low intensity resistance load while still offering the potential to generate an equivalent increase in muscular strength as the advised resistance training regimens with relatively high loads. Beneficial physiological effects of BFRT include recruitment of type II muscle fibers and stimulation of growth hormone activity induced by partial vascular occlusion, in addition to its mechanical advantages. BFRT procedure paired with low intensity resisted exercise are proposed to cause pain modulation by exercise induced hypoalgesia (EIH) and conditioned pain modulation (CPM) pathways by activating descending pain inhibition.

Blood flow restriction (BFR) training has emerged as a promising technique that has shown potential benefits in enhancing muscular strength and promoting tissue adaptation without subjecting the body to high-intensity exercises. ^3^ Despite its applications in various musculoskeletal conditions, the potential efficacy of BFR training in managing persistent neck pain remains relatively unexplored. This study protocol aims to investigate the effect of blood flow restriction training on pressure pain threshold and hand function among adults suffering from persistent neck pain. Utilizing a randomized controlled trial design, this research seeks to provide valuable insights into the role of BFR training as a potential therapeutic option for this challenging and often refractory condition. The rationale for examining pressure pain threshold lies in its significance as a common clinical measure for assessing pain sensitivity and tolerance, particularly in musculoskeletal pain conditions. ^4^ Understanding the impact of BFR training on pressure pain threshold will offer valuable information on the technique’s ability to modulate pain perception in individuals with persistent neck pain. Additionally, assessing hand function is of paramount importance, as neck pain can often result in functional limitations that affect daily activities and work performance. ^5^ Investigating the effects of BFR training on hand function will shed light on whether this intervention can lead to functional improvements and enhance the overall well-being of individuals experiencing neck pain. The results obtained from this research hold the potential to inform healthcare practitioners, physiotherapists, and other relevant professionals about the feasibility and effectiveness of incorporating BFR training into the management strategies for individuals with persistent neck pain. By addressing the current research gap and exploring the impact of BFR training on pressure pain threshold and hand function, this study protocol aspires to contribute to evidence-based practices in pain management and rehabilitation, ultimately enhancing the quality of life for those affected by persistent neck pain.

## Objectives

I. To evaluate the effect of BFRT in alleviating neck pain and improving the hand function among adults with persistent neck pain.
II. To study the impact of BFRT on activities of daily living and quality of life among patients with persistent neck pain.
III. To study the long term effect of BFRT on precision and power grip among adults with persistent neck pain.

## Protocol

### Study design

This study will be a prospective 1:1 allocation, parallel group active controlled trail conducted at Physiotherapy Department, Galgotias University *.* Ethical clearance has been obtained from Departmental Ethics Committee on 19/05/2023 with reference number DEC/PT/GU/2023 and the trial was prospectively registered with the Clinical Trial Registry India CTRI/2023/06/053439. Informed consent will be obtained from all the participants who are eligible to be included in the study.

### Null hypothesis

There is no difference between the BFRT and conventional physiotherapy group in alleviation of pain, and hand function among adults with neck pain.

### Alternate hypothesis

BFRT will have a significant impact in reducing pressure pain threshold and improving hand function in patients with persistent neck pain among adults.

***Subjects***

**Figure 1.**
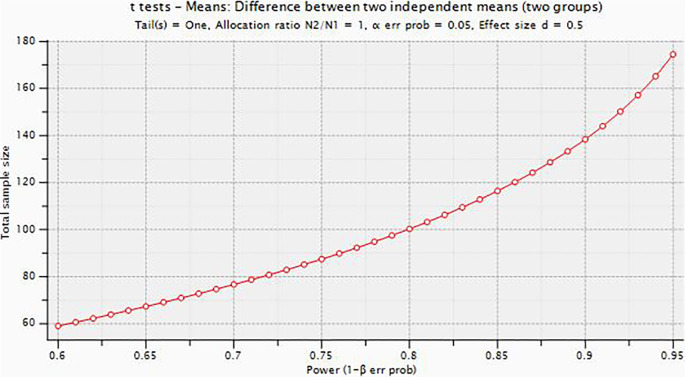
Plot describing the total sample size with power (1-β).

**Figure 2.**
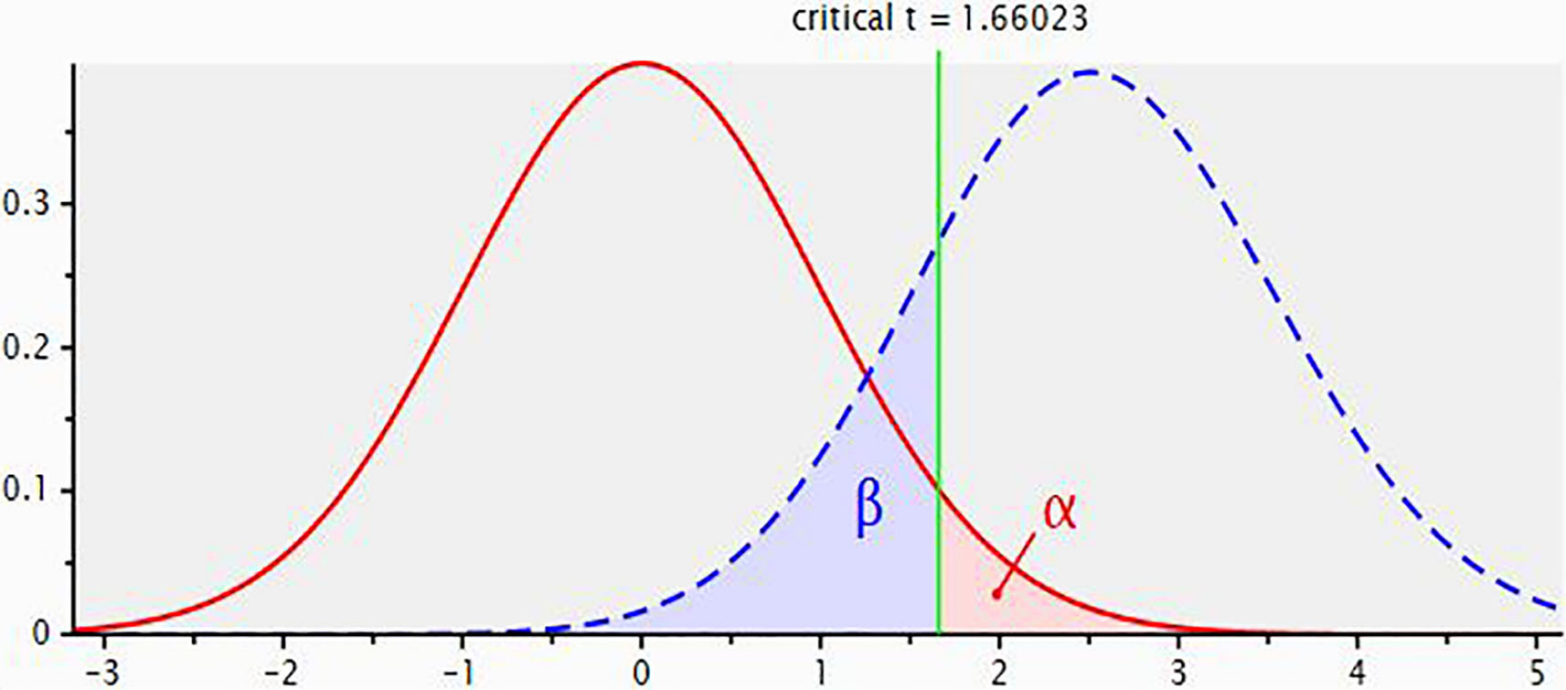
Plot showing central and non-central distributions.

One hundred and ten self-reporting persistent neck pain for more than three months duration will be screened for eligibility and recruited in the study. After obtaining informed consent, the participants will be randomly allocated in 1:1 ratio to the BFR training group and the control group using a computer-generated randomization sequence. Allocation concealment will be ensured to maintain blinding and reduce potential bias. Sample size was calculated using Gpower version 3.1.9.4 for Windows by assuming effect size of 0.5(Cohen’s D medium size effect was assumed) with the margin of error (α) to test the level of significance was set at 0.05 and (1-α) 80% βpower and confidence interval at 95%, the final derived sample with added 10% of contingency was n=112 ^6^ ( Figure 1). See Figure 2 for central and non-central distributions.

### Eligibility criteria

Participants aged 18 and above years, both male and females, and those who self-reported non-specific neck pain for more than 3 months duration, and also present with weak power grip and/or impaired hand precision functions, and those who will be able to prospectively visit the study center will be invited to participate in the trial.

### Exclusion criteria

The exclusion criteria will be patients those who have history of receiving BFRT, deformity of upper extremity, spinal deformity, unhealed injuries in spine and upper extremity, uncontrolled hypertension, history of deep vein thrombosis and medically diagnosed neurological conditions that interfere with hand function or causes pain.

## Methods

Written informed consent will be obtained from all the participants who are eligible to be included in the study. The diagnosis of persistent neck pain will be made based on previous history and duration. Patients with radiating pain to shoulders or hands will be excluded. Enrolment, intervention, and assessment will be done as per the SPIRIT guideline ( Table 1). Participants will be obtained from the OPD of Physiotherapy Department and from all over the university including the teaching, non-teaching staff, from camps nearby and students. Ethical clearance has been obtained from Departmental Ethics Committee on 19/05/2023 with reference number DEC/PT/GU/2023 and the Trial has been registered with Clinical Trial Registry India CTRI/2023/06/053439. All patients diagnosed with chronic neck pain and weak hand function will sign the informed consent. Demographic data, history and other data will be obtained at the baseline. The study will be done in accordance to the declaration of Helsinki. ^7^

**Table 1.** Schedule of enrolment, Intervention, and assessment, the SPIRIT guidelines.

Study period	Screening	Baseline (T0)	Training Sessions			Intervention Period in weeks								Post interventions Assessment (T1)	Follow up T2
Time (Weeks)	-4 to -1		S1 to S7	S8 to S16	S17 to S24	1	2	3	4	5	6	7	8	x	x
Patient Data	**x**														
Informed Consent	**x**														
Demographic Data	**x**														
Medical History	**x**														
Allocation		**x**													
Control group		**x**				**x**	**x**	**x**	**x**	**x**	**x**	**x**	**X**		**x**
Intervention group		**x**				**x**	**x**	**x**	**x**	**x**	**x**	**x**	**x**		**x**
Pressure pain Threshold		**x**												**x**	**x**
Hand Function		**x**												**x**	**x**
WHO5 INDEX		**x**												**x**	**x**
Compliance and motivation			**x**	**x**	**x**									**x**	
Patient Diary		**x**	**x**	**x**	**x**	**x**	**x**	**x**	**x**	**x**	**x**	**x**	**x**		
Routine PT	**X**	**x**													
Adverse Effects			**x**	**x**	**x**	**x**	**x**	**x**	**x**	**x**	**x**	**x**	**x**		

Note: The ‘x’ mark affirms the event, S – session, T0 – Baseline, T1 – Post-intervention recording, T2 – follow-up recording.

### Intervention

All patients will receive standard physiotherapy care. It will include thermo therapy, neck isotonic exercises. The Experimental group will also receive BFR with single session with 75 vol LOP 40 to 50 % for upper grip ball exercise (Light resistance) wrist curls and wrist extension using long leavers Flexibar radial and ulnar deviation. Follow up will be done for the patients on second visit after 8 weeks and long-term follow up after 6 months for others on phones who fail to come for follow up. All the patients will be explained about the possible benefits and complete procedure of the study and consent will be signed by each patient who is willing to participate in the study. They will be told its voluntary and they can withdraw anytime from the study and it won’t affect their treatment if they refuse to be part of the study. Patient information will be kept confidential. Each individual will serve as self-control to report about the possible benefit in pain score after receiving BFRT and after follow up. See Table 2 for the full BFRT protocol. ^8^

**Table 2.** Protocol of BFR.

Mode of exercise prescription with BFR	
**Frequency**	2-3 times a week for 8 weeks
**Load**	20-40% 1RM
**Restriction time**	5-10 min per exercise (reperfusion between exercises)
**Type**	Small and large muscle groups (arms and legs/unilateral or bilateral)
**Sets**	2-4
**Cuff**	5 (small,) 10x 12 (medium), 17 or 18 (large)
**Repetitions Pressure**	(75 reps) – 30 x 15 x 15, or sets to failure 40-80 % AOP
**Rest between sets**	30-60 seconds
**Restriction form**	Continuous or intermittent
**Execution Speed**	1-2 (concentric or eccentric)
**Execution**	Until concentric failure or when planned rep scheme is completed

### Outcome measures

***Pressure pain threshold using Algometer***

Using an algometer, a portable instrument that measures pressure sensitivity at particular anatomical locations in the neck region, the pressure pain threshold (PPT) will be determined. The algometer consists of a gauge with a pressure-sensing tip attached that measures applied pressure in kilopascals (kPa). Before the assessments begins, the algometer will be calibrated to ensure accuracy and consistency of measurements. Participants will be comfortably seated in an upright position with their neck muscles relaxed. It is essential to explain the procedure to the participant, ensuring they understand the assessment and can provide feedback on their pain perception. Specific anatomical points in the neck region will be chosen for the assessment. Commonly, these points include the trapezius, levator scapulae, and sternocleidomastoid muscles. The researcher will progressively apply pressure to the selected spots using the algometer. A button must be pressed to cease the pressure application or the participant must verbally report feeling pain for the pressure to increase at a constant rate (for example, 1 kPa/s).

***Neck Outcome Score***

The Neck Outcome Score (NOS) is a self-reported questionnaire that assesses various aspects of neck pain and its impact on daily activities and quality of life. Participants will be provided with the NOS questionnaire, and the research team will explain its purpose and how to complete it. It’s important to ensure that participants understand the questions and how to score their responses accurately. The NOS typically includes questions related to pain intensity, pain-related disability, functional limitations, and the impact of neck pain on quality of life.

***Grip strength using hand help dynamometer***

Grip strength will be measured using a handheld dynamometer. We will ensure that the participant is well-rested and not fatigued before the measurement. We will also inform the participant about the purpose of the grip strength measurement and the procedure involved. The dynamometer will be adjusted to fit the participant’s hand size. The participant will be seated on a chair without armrests, ensuring proper posture with their back straight, feet flat on the ground, and forearm resting on a flat surface. We will instruct the participant to hold the dynamometer in their dominant hand. When the participant is ready, they should be instructed to squeeze the dynamometer handle with maximum effort for about 3 to 5 seconds. The researcher can provide encouragement to the participant during the measurement to ensure they exert their maximum effort. The highest grip strength measurement achieved during the trial will be recorded.

***WHO-5 quality of life index questionnaire***

The WHO-5 quality of life index questionnaire is used to assess an individual’s subjective well-being and quality of life. We will briefly explain the purpose of the WHO-5 questionnaire to the participant and let them know that it’s designed to assess their emotional well-being and quality of life. Participants will rate their agreement with each statement on a scale from 0 to 5, where 0 indicates “at no time” and 5 indicates “all of the time”. After participants have provided their responses, we can assist them in calculating their total score. A higher score on the WHO-5 indicates better emotional well-being and quality of life.

### Statistical analysis

Data will be analysed using IBM Statistical Package for Social Sciences version 21 for Windows. The patient characteristics at the baseline will be presented as mean, standard deviation and proportion with 95% confidence interval. The kolmogorov-smirnov and Shapiro Wilk test will be performed to assess the normality distribution of all the outcome scores. The student’s t- test and paired t-test will be used to compare between the groups and intra group and multiple regression analysis will be done to check for any correlation or any factor influencing the outcomes of the study.

## Discussion

The present study protocol presents a RCT testing the effectiveness of BFRT on pressure pain threshold and hand function among adults with persistent neck pain. The intervention aims to reduce pain and hand function by the proposed mechanism. The study intends to employ a randomized controlled trial design, ensuring scientific rigor and minimizing bias. By comparing the BFR training intervention group with a control group, researchers seek to assess the impact of this innovative technique on two primary outcomes: pressure pain threshold and hand function. Therefore, we expect that the adults with neck pain and compromised hand function in the BFRT group will exhibit improvement in terms of pain and hand function in comparison to the neck pain patients in the conventional group. The significance of this research lies in its potential to contribute valuable insights into managing neck pain and improving hand function using a novel approach like BFR training. If the results show positive effects, it could pave the way for a non-invasive, safe, and cost-effective intervention for individuals dealing with persistent neck pain. Ultimately, this study may have a meaningful impact on the quality of life for those suffering from this prevalent issue, offering hope for improved pain management and functional recovery.

**Study Status**: At the moment the authors are involved in training the assessors and physiotherapists those who will be involved in interventions and we are planning to start screening after one month.

### Social and research benefit

The chronic neck pain may progress to cause disability and diminished quality of life. A non-invasive timely intervention may cause halting adverse effects of central sensitization in patients suffering chronic neck pain. A simple and novel intervention may be incorporated.

## Data Availability

No data are associated with this article.
