# Supplementary material for: Effect of blood flow restriction training on pressure pain threshold and hand function among adults with persistent neck pain: A study protocol for a randomized controlled trial
Source: F1000Res. 2024 May 31;12:1076. Originally published 2023 Aug 31. [Version 2] doi: 10.12688/f1000research.140084.2 (PMC11165297; doi:10.12688/f1000research.140084.2)
Supplement: Peer review history

# Reviewer report: Reviewer response for version 2

**DOI:** 10.5256/f1000research.167162.r285392

**Published:** 2024-06-10

**Authors:** Yujiro Yamada

**Affiliations:** 1 The University of Mississippi, Oxford, Mississippi, USA

Once again, thank you for choosing me as a reviewer for this manuscript. I appreciate the time and effort that the authors put into revising the manuscript. 

 The introduction has improved from the first draft by addressing other reviewer's comments; however, there are still many ambiguities in the method and statistical analyses, which I have mentioned in the initial comments. Please consider addressing my comments and provide revisions as needed. I would also appreciate it if authors could provide rebuttals to any of my comments which are not sufficient enough to address. Please let me know if my initial comments are not clear. I am happy to reiterate the sentences.

Is the study design appropriate for the research question?

Partly

Is the rationale for, and objectives of, the study clearly described?

Partly

Are sufficient details of the methods provided to allow replication by others?

No

Are the datasets clearly presented in a useable and accessible format?

Not applicable

Reviewer Expertise:

Blood flow restriction, resistance training, pressure pain threshold, blood pressure measurements, strength assessments, ultrasound muscle thickness assessment, and cognitive function assessment.

I confirm that I have read this submission and believe that I have an appropriate level of expertise to confirm that it is of an acceptable scientific standard, however I have significant reservations, as outlined above.

# Reviewer report: Reviewer response for version 2

**DOI:** 10.5256/f1000research.167162.r285391

**Published:** 2024-06-03

**Authors:** Imam Subadi

**Affiliations:** 1 Universitas Airlangga, Surabaya, Indonesia

I'd like to thank the author and team for the considerable effort and attention to detail put into revising this manuscript following the initial review.

 The clarity of arguments, methodology, and overall presentation have markedly improved.

Is the study design appropriate for the research question?

Yes

Is the rationale for, and objectives of, the study clearly described?

Yes

Are sufficient details of the methods provided to allow replication by others?

Partly

Are the datasets clearly presented in a useable and accessible format?

Partly

Reviewer Expertise:

wet cupping therapy, pain, stroke

I confirm that I have read this submission and believe that I have an appropriate level of expertise to confirm that it is of an acceptable scientific standard.

# Response

**Published:** 2024-06-04

**Authors:** Dr Mohammad Sidiq

Thank you so much for the review. We are grateful for the time you took to review our paper.

# Reviewer report: Reviewer response for version 1

**DOI:** 10.5256/f1000research.153413.r275252

**Published:** 2024-05-27

**Authors:** Yujiro Yamada

**Affiliations:** 1 The University of Mississippi, Oxford, Mississippi, USA

General Comments:

 The authors of the manuscript proposed the study protocol to investigate the effect of adding blood flow restriction (BFR) training to standard physiotherapy care on pressure pain threshold in the neck region and hand function in adults with persistent neck pain, using a randomized controlled trial design. I would think this study idea would add valuable insight into the literature on BFR and pain. However, I have multiple parts that are not clear in the manuscripts, especially in the protocol section. Also, there is a suggestion of using a prospective mediation analysis rather than or in addition to the multiple correlation analysis. I would like the authors to consider these comments and provide revisions or reasonable rebottle accordingly. Thank you for the opportunity to review this paper.

 Comments Specific to Individual Sections:

 ABSTRACT

 Comments for Authors

- It is not clear in the abstract that the BFR training group also will receive standard care, which is an important narrative that needs since BFR training + standard care vs. standard care is different from BFR training vs. standard care.

INTRODUCTION

 Comments for Authors

- Thank you for the concise introduction.
- “…subjecting the body to **high-intensity** exercises.”
  - I would avoid using “high intensity” in the context. It would be rather suitable to use “high-load” since high intensity could also refer to how much “effort” the individuals put in during the exercise (Steele 2014, [Ref 1]). For instance, performing the “low-load resistance exercise” can be a “high-effort” exercise when the exercise is taken near or to momentary failure.
- “Despite its application in various musculoskeletal conditions”
  - This sentence acknowledges that there are studies examining the effect of BFR training on pain reduction; however, it is unclear whether BFR training worked for these conditions and no citation was provided. For instance, a review article summarized the acute and chronic effects of BFR exercise on pain reduction, and some included studies showed the greater pain-reducing effect of BFR training compared to high-load resistance training in adults after ACL (Song JS, et al., 2021 [Ref 2]). No need to be this paper, but please consider citing a work for this part of the sentence.
- I am not sure I understand, but the authors noted that neck pain can result in functional limitations that affect daily activities and work performance. Is there any relationship between neck pain and grip strength at baseline or any evidence or physiological rationale that changing one leads to change in another? If so, the authors could propose to perform mediation analysis, which tests whether the improvement in grip strength occurs through the potential greater pain reduction in BFR training than in standard care. Different from the correlation analysis, this mediation analysis can test the causal relationship by using the rigorous linear regression approach. You might consider this approach and this paper as guidance (Valente MJ, et. al., 2017 [Ref 3])

PROTOCOL

- “One hundred and ten self-reporting persistent neck pain…”
  - Would the authors consider the number of participants who are screened out due to the exclusion criteria the number of dropouts, or the inability to follow-up? Considering these concerns, would authors be better to shoot for higher than 110 (possibly 120) to have adequate power to detect the moderate effect?
- “weak power grip, impaired hand precision function”
  - How will the authors determine “weak handgrip, impaired hand function”? Please provide reasonable inclusion criteria for the relatively weak handgrip and function for the target population.
- “75 vol LOP 40 5o 50% for upper grip ball exercise (light resistance wrist curls) …
  - Please describe the BFR exercise session more precisely and clearly. What does the 75 vol mean? Meaning 4 sets of 30, 15, 15, 15 repetitions? What blood flow restriction device will the author be using? Hokanson? Delfi? Smart cuff? Or other devices? How would you determine limb occlusion pressure (LOP)? How would the authors determine the load for the grip ball exercise? How are the authors performing one repetition maximum (1RM) test on the movement? Only at the first visit? Table 2 will leave me with many questions about the exercise protocol. This table is very similar to Table 1 of Patterson et al. 2019, which is the guideline paper for BFR. Please be specific about the protocol. The load, 20 – 40% 1RM: Does it mean the exercise is going to be progressive from 20 – 40% 1RM? If so, how would the load be progressed (like a 5% increase every other week?). The cuff size: Which cuff would the author use for the exercise, small, medium, or large cuff? The length can differ depending on the size of the participants; however, the cuff width should be consistent since it affects the arterial occlusion pressure and possible discomfort. Repetitions & pressure: please report the number of repetitions completed in each set when reporting the results in the future since some participants might not be able to complete the predetermined repetitions. For pressure, is the author progressing the pressure as well from 40 – 80% AOP? Why does it say AOP but in the text it says LOP? Please be consistent throughout the manuscript. Same as rest periods…, if the author gives the range, please specify whether the rest period would be progressed to be shorter from 60 to 30 seconds or rather specify the rest period. Same comment for the exercise speed.
- “Follow up will be done for patients on the second visit after 8 weeks and long term follow up after 6 months for others on phones who fail to come for follow up”
  - Please clarify the sentence. Please revise the second visit as a “post-training visit” to avoid confusion from readers. This reads as if the participants cannot come to the post-training visit, they will be followed up by phone after 6 months, which does not make sense to me.
- “Each individual will serve as self-control to report about the possible benefit in pain score after receiving BFR and after follow up”
  - It is a good idea to report the participants’ expectations about the intervention in both groups at baseline and post-intervention, and 6-month follow-up. But, I don’t think it is correct to say “self-control” in this context. I do not understand how this variable will be used as “self-control.”
- Please clarify what parts are supervised and unsupervised in the intervention.
- Please give a more specific description of the anatomical points in the neck region or provide a diagram or picture for clarity.
- For statistical analysis, the authors may consider reporting the variables as median if the variables are not normally distributed.
- As mentioned, in the introduction, mediation analysis could give better insight into the potential relationship between the outcome variables rather than multiple correlation analysis. If the author wants to report the correlation analysis, please specify which variables will be used for the analysis.

Is the study design appropriate for the research question?

Partly

Is the rationale for, and objectives of, the study clearly described?

Partly

Are sufficient details of the methods provided to allow replication by others?

No

Are the datasets clearly presented in a useable and accessible format?

Not applicable

Reviewer Expertise:

Blood flow restriction, resistance training, pressure pain threshold, blood pressure measurements, strength assessments, ultrasound muscle thickness assessment, and cognitive function assessment.

I confirm that I have read this submission and believe that I have an appropriate level of expertise to state that I do not consider it to be of an acceptable scientific standard, for reasons outlined above.

## References

[1] : Intensity; in-ten-si-ty; noun. 1. Often used ambiguously within resistance training. 2. Is it time to drop the term altogether?. *Br J Sports Med* .2014;48(22) : 10.1136/bjsports-2012-092127 1586-8 10.1136/bjsports-2012-092127 23403526

[2] : Exercise-induced hypoalgesia and pain reduction following blood flow restriction: A brief review. *Phys Ther Sport* .2021;50: 10.1016/j.ptsp.2021.04.005 89-96 10.1016/j.ptsp.2021.04.005 33940556

[3] : Comparing models of change to estimate the mediated effect in the pretest-posttest control group design. *Struct Equ Modeling* .2017;24(3) : 10.1080/10705511.2016.1274657 428-450 10.1080/10705511.2016.1274657 28845097 PMC5568008

# Reviewer report: Reviewer response for version 1

**DOI:** 10.5256/f1000research.153413.r275253

**Published:** 2024-05-16

**Authors:** Imam Subadi

**Affiliations:** 1 Universitas Airlangga, Surabaya, Indonesia

REVIEW

 This paper aimed to evaluate the effects of blood flow restriction training on pressure pain threshold and hand function among adults with persistent neck pain. There are major concerns that need to be addressed by the authors.

 Abstract :

 The authors don't need to mention ethical clearance in the abstract

 Introduction:

1. The importance, role and mechanism of blood flow restriction training is not well highlight in this section as a therapeutic potential in neck pain and hand function 
2. Which part of the pain pathway is targeting ?
3. The reason for choosing the blood floe restriction training to reduce pain and increase hand function is not well explained

Objective :

 The objective of the study must be inline with the title. The author mentioned the impact of BRT on disability. How the disability is measured ?

 Methods

1. The determination of the diagnosis is not clear ? Who diagnosed the patients ?
2. In table 1, the author must be explain the abbreviations
3. Intervention : How is the author determine 1 RM?
4. In table 2 : How is the author determine restriction form  continuous or intermittent ?

Is the study design appropriate for the research question?

Yes

Is the rationale for, and objectives of, the study clearly described?

Yes

Are sufficient details of the methods provided to allow replication by others?

Partly

Are the datasets clearly presented in a useable and accessible format?

Partly

Reviewer Expertise:

wet cupping therapy, pain, stroke

I confirm that I have read this submission and believe that I have an appropriate level of expertise to confirm that it is of an acceptable scientific standard, however I have significant reservations, as outlined above.

# Reviewer report: Reviewer response for version 1

**DOI:** 10.5256/f1000research.153413.r222257

**Published:** 2023-11-15

**Authors:** Aminu A Ibrahim

**Affiliations:** 1 Department of Physiotherapy, School of Basic Medical Sciences, Skyline University Nigeria, Kano, Nigeria

**General comments:**

 The manuscript aims to investigate the potential benefits of Blood Flow Restriction Training (BFRT) as a treatment intervention for persistent neck pain. The authors need to revise the entire manuscript for grammar and technicalities. I have the following comments for the authors.

**Abstract**

1. Under methods, replace done with ‘ to be conducted’
2. The authors don’t need to mention informed consent in the abstract
3. 110 patients should be written in words
4. In reporting study protocol, no need to have a results section
5. The conclusion should be revised for grammar.

**Introduction, objectives, and hypotheses**

1. The rationale for evaluating BFR training for neck needs to be more explicit
2. The authors focused more on highlighting the potential benefit of the new intervention, which could turn out to be as effective as the standard care.
3. This is a protocol, the write-up should be presented in the future tense
4. The objectives are not clearly outlined. What about the comparison between the BFR training and standard care? The authors mentioned long-term, what about short-term?
5. I would also advise the authors to evaluate outcomes at 4 weeks
6. Hypotheses are not well constructed and no need to add the null hypothesis in the article write-up. The heading should just read "Hypotheses" The hypotheses should indicate the comparison between the groups.

**Methodology**

1. The authors mentioned 6 months as long term, this is often considered to be intermediate term. Long-term periods typically involved 12 months or 1 year or above
2. The authors mentioned that participants will be assigned into two groups equally but the type of randomization to achieve this equal allocation (i.e. block randomization with the block sizes) is not mentioned.
3. Figures 1 and 2 for sample size calculations are not needed. Please consider deleting
4. Why was the effect size of 0.50 selected? The authors have not made it clear if the primary outcome - pressure pain threshold or hand function has been used in the determination of effect size assumptions. Please clarify.
5. The reliability of using Algometer for measuring Pressure pain threshold and a hand help dynamometer for measuring hand function should be reported
6. I suggest the authors should also evaluate the intensity of neck pain using the Numerical Pain Rating Scale or Visual Analogue Scale.
7. Reliability (ICC and Cronbach’s alpha) and validity for the Neck Outcome Score (NOS) and WHO-5 quality of life index questionnaire should be briefly reported.

Is the study design appropriate for the research question?

Yes

Is the rationale for, and objectives of, the study clearly described?

Yes

Are sufficient details of the methods provided to allow replication by others?

Partly

Are the datasets clearly presented in a useable and accessible format?

Not applicable

Reviewer Expertise:

Musculoskeltal disorders, clinical epidemiology, cross-cultural research

I confirm that I have read this submission and believe that I have an appropriate level of expertise to confirm that it is of an acceptable scientific standard, however I have significant reservations, as outlined above.

# Response

**Published:** 2023-11-27

**Authors:** Dr Mohammad Sidiq

**Methodology**

1. The authors mentioned 6 months as long term, this is often considered to be intermediate term. Long-term periods typically involved 12 months or 1 year or above
2. The authors mentioned that participants will be assigned into two groups equally but the type of randomization to achieve this equal allocation (i.e. block randomization with the block sizes) is not mentioned.
3. Figures 1 and 2 for sample size calculations are not needed. Please consider deleting
4. Why was the effect size of 0.50 selected? The authors have not made it clear if the primary outcome - pressure pain threshold or hand function has been used in the determination of effect size assumptions. Please clarify.
5. The reliability of using Algometer for measuring Pressure pain threshold and a hand help dynamometer for measuring hand function should be reported
6. I suggest the authors should also evaluate the intensity of neck pain using the Numerical Pain Rating Scale or Visual Analogue Scale.
7. Reliability (ICC and Cronbach’s alpha) and validity for the Neck Outcome Score (NOS) and WHO-5 quality of life index questionnaire should be briefly reported.

**Reviewer  Comments:**

**Author Responses:**

**Abstract**

  

1.Under methods, replace done with ‘ to be conducted’
2.The authors don’t need to mention informed consent in the abstract
3.110 patients should be written in words
4.In reporting study protocol, no need to have a results section
5.The conclusion should be revised for grammar.

**Background**: Persistent neck pain is a prevalent musculoskeletal condition that affects the quality of life and functional abilities of individuals. Blood Flow Restriction Training (BFRT) is a novel therapeutic approach that involves restricting blood flow to exercising muscles to enhance strength and function. However, limited research has been conducted on the effects of BFRT on pressure pain threshold and hand function in adults with persistent neck pain. This randomized controlled trial aims to investigate the potential benefits of BFRT as a treatment intervention for this population.

**Methods: **This study will be a prospective parallel group active controlled trail to be conducted at Physiotherapy Department, Galgotias University *. *Ethical clearance has been obtained from Departmental Ethics Committee on 19/05/2023 with reference number DEC/PT/GU/2023 and the Trial has been registered with Clinical Trial Registry India CTRI/2023/06/053439. One hundred and ten patients with persistent neck pain will be randomly allocated into two groups. The BFRT group will receive supervised training sessions three times a week for eight weeks, performing low-load resistance exercises with blood flow restriction applied using personalized cuff pressure. The control group will receive standard care for neck pain, which may include general advice, manual therapy, and/or home exercises without BFRT. The primary outcome measures will be the pressure pain threshold, assessed using a pressure Algometer, and hand function, evaluated using standardized tests such as Hand Grip Strength and Purdue Peg board Test.

**Conclusion: **This study aims to provide significant insights into the potential advantages of BFR training for enhancing pressure pain threshold and hand function among adults experiencing persistent neck pain.

**Methodology**

 The rationale for evaluating BFR training for neck needs to be more explicit

 Recent research has offered insights indicating that both low-load training until volitional fatigue and low-load blood flow restriction (BFR) training have the potential to induce muscle size gains comparable to high-load training.

 The authors focused more on highlighting the potential benefit of the new intervention, which could turn out to be as effective as the standard care.

 Thank you for your thoughtful feedback. We appreciate your observation regarding our focus on highlighting the potential benefits of the new intervention in comparison to standard care. We acknowledge the importance of addressing this concern and will make sure to provide a more balanced discussion in our revised manuscript. We will include a more comprehensive analysis that considers the effectiveness of both the new intervention and standard care to ensure a thorough and well-rounded presentation of our findings.

 This is a protocol, the write-up should be presented in the future tense

 The results obtained from this research might hold the potential to inform healthcare practitioners, physiotherapists, and other relevant professionals about the feasibility and effectiveness of incorporating BFR training into the management strategies for individuals with persistent neck pain.

 The objectives are not clearly outlined. What about the comparison between the BFR training and standard care? The authors mentioned long-term, what about short-term?

**Revised Objectives are**

 To evaluate the potential effect of BFRT in alleviating neck pain and improving the hand function among adults with persistent neck pain.

 To study the impact of BFRT on disability and quality of life among patients with persistent neck pain

 To study the short-term effect of BFRT on Precision and Power grip among adults with Persistent neck pain.

  

1. I would also advise the authors to evaluate outcomes at 4 weeks

**We have revised the outcomes at four weeks and a follow up after twelve weeks.**

  

1. Hypotheses are not well constructed and no need to add the null hypothesis in the article write-up. The heading should just read "Hypotheses" The hypotheses should indicate the comparison between the groups.

**Revised Hypotheses are as under**

1. BFRT might have a significant impact in reducing pressure pain threshold and improving hand function in patients with persistent neck pain among adults.
2. BFRT will demonstrate a statistically significant increase in pressure pain threshold and improvement in hand function compared to a control group receiving standard care.
3. To study the short-term effect of BFRT on Precision and Power grip among adults with Persistent neck pain

**Methodology**

 The authors mentioned 6 months as long term; this is often considered to be intermediate term. Long-term periods typically involved 12 months or 1 year or above

**The short term follow up will be done after 6 months due to feasibility constraints and social factors of participants.**

 The authors mentioned that participants will be assigned into two groups equally but the type of randomization to achieve this equal allocation (i.e. block randomization with the block sizes) is not mentioned.

**To ensure equal allocation across group’s overtime, a block randomization with the block size of 8 will be used.**

 Figures 1 and 2 for sample size calculations are not needed. Please consider deleting

**Yes we agree, thank you**

 Why was the effect size of 0.50 selected? The authors have not made it clear if the primary outcome - pressure pain threshold or hand function has been used in the determination of effect size assumptions. Please clarify

**Assuming that BFRT might have an medium effect on the variable pressure pain threshold,  the Cohen’s d effect of 0.5 for medium effect was used.**

**Sample size was calculated for the variable pressure pain threshold assessed by algometer using G power version 3.1.9.4 for Windows. A priori power analysis with the assumed Cohen’s d medium effect size of 0.5, with the margin of error (α) to test the level of significance, power of study (1 – β) was set at 80%, and the confidence interval at 95%. Assuming a drop out of up to 10%, a target sample of n= 112 was estimated.**

 The reliability of using Algometer for measuring Pressure pain threshold and a hand help dynamometer for measuring hand function should be reported

**The hand-held algometer demonstrated a reliability of r 0.990** [Bala J1] **for average force application of 80 Newton’s. and the Jamar hand-held dynamometer showed a reliability of 0.94** [Bala J2] **.**

 I suggest the authors should also evaluate the intensity of neck pain using the Numerical Pain Rating Scale or Visual Analogue Scale.

**Since, one of the sub-domain of the Neck Outcome Score (NOOS) measure the pain intensity, we thought that would address the self-reported pain intensity.**

  

1. Reliability (ICC and Cronbach’s alpha) and validity for the Neck Outcome Score (NOS) and WHO-5 quality of life index questionnaire should be briefly reported.

**The Neck Outcome score (NOOS) subscales was reported to have a good to excellent reliability (ICC 0.88 – 0.95** [Bala J3] **) among participants with neck pain. Further, NOOS was found to have a minimal detectable changes ranging between 1.1 and 1.9. The WHO-5 wellbeing index showed an acceptable internal consistency of 0.79 among Indian adults** [Bala J4] **.**

 Kinser, Ann M ^1^; Sands, William A ^1^; Stone, Michael H ^2^. Reliability and Validity of a Pressure Algometer. Journal of Strength and Conditioning Research 23(1):p 312-314, January 2009. | DOI: 10.1519/JSC.0b013e31818f051c

  Benton MJ, Spicher JM, Silva-Smith AL. Validity and reliability of handgrip dynamometry in older adults: A comparison of two widely used dynamometers. Plos one. 2022 Jun 21;17(6):e0270132.

 Juul T, Søgaard K, Davis AM, Roos EM. Psychometric properties of the Neck OutcOme Score, Neck Disability Index, and Short Form–36 were evaluated in patients with neck pain. Journal of clinical epidemiology. 2016 Nov 1;79:31-40.

  Pattnaik P. Validation of the World Health Organisation 5-Item Well-Being Index (WHO-5) among the Adult Population Living in a Chronically Arsenic Affected Area of Rural West Bengal in India. Indian Journal of Public Health Research & Development. 2020 Mar 1;11(3).
